# “Don’t leave us behind”: a qualitative study exploring the feasibility of a palliative care training program for non-health caregivers in Honduras

**DOI:** 10.1177/26323524251316897

**Published:** 2025-02-06

**Authors:** Sheryl Ruiz, Martin Stafström, Leda Parham, Luis Orellana

**Affiliations:** Faculty of Medicine, Lund University, Jan Waldenströms gata 35, Malmö 21428, Sweden; Department of Social Medicine and Global Health, Lund University, Malmö, Sweden; Department of Biology and Department of Nursing, Universidad Nacional Autónoma de Honduras–UNAH Campus Atlántida, La Ceiba, Honduras; Department of Nursing, Universidad Nacional Autónoma de Honduras–UNAH Campus Atlántida, La Ceiba, Honduras

**Keywords:** caregiver, cuidados paliativos, family dynamics, feasibility, health determinants, health equity, Honduras, intersectoral collaboration, palliative care, qualitative study

## Abstract

**Background:**

Palliative care (PC) can alleviate suffering and improve quality of life. Yet, disparities persist, particularly in Honduras. Training efforts in PC for non-health caregivers have proven to reduce burnout and stress while enhancing the quality of life for both caregivers and patients.

**Purpose:**

This study aimed to explore the feasibility of a PC training program for non-health caregivers in Honduras.

**Methods:**

This exploratory study utilized latent content analysis within an interpretivist paradigm. Data was collected through individual semi-structured interviews with 25 participants belonging to different key groups: patients diagnosed with cancer, non-health caregivers, PC experts, and health-related decision makers. Interviews were conducted in Honduras’ three main cities: Tegucigalpa, San Pedro Sula, and La Ceiba. The sampling technique employed was maximum variation.

**Results:**

PC patients and caregivers face significant emotional challenges, often worsened by poverty and resource limitations, which leads to a strain in family dynamics. A lack of education, driven by inadequate healthcare education and policies, contributes to widespread misconceptions about PC. However, progress through various sectors aligned with the same goals proves Honduras is a country with potential. A program with a bottom-up approach, with intersectoral collaboration and training tailored to the needs of caregivers and patients, is urgently needed.

**Conclusion:**

It highlights the feasibility, necessity, and potential replicability of implementing a PC training program for non-health caregivers in Honduras, which could offer significant benefits at both individual and national levels. A program that truly accounts for health determinants may help mitigate PC shortages in similar regions. Further research and policy advocacy are essential to empower underserved populations and foster health equity across low- and middle-income settings, to ensure no one is left behind.

## Background

Annually, more than 56.8 million individuals need palliative care (PC).^1^ PC prevents and alleviates suffering of any kind, enhancing the life quality for patients and families facing life-threatening illnesses.^2^ Though it is an essential part of health,^3–5^ only 1 in 10 people actually receive it.^2^

Only 15% of countries worldwide have achieved advanced integration of PC. Most of these countries are concentrated in Europe, such as Sweden, Denmark, and Germany. In Latin America, Costa Rica is the country that stands out as it is at the same level of advanced integration as European countries.^1^ This is concerning since 3.4 million Latin Americans are suffering from a serious illness each year.^6^ This is particularly true in Honduras, a country where PC services receive little priority.^7^

Despite that 46,000 Hondurans are suffering from serious illnesses including 8,800 suffering from cancer,^6^ the country ranks among those in Latin America with the greatest shortage of PC services, reporting 0.64 available services per million residents in 2020.^8^ That same year, only eight countries in Latin America reported offering academic training in PC, and Honduras was not one of them.^8^ At that time, PC was also not covered by the country’s National Health System. As a result, there was no dedicated budget, policy framework, or systematic development for its delivery or research.^7,8^ Furthermore, in Honduras, opioids equivalent to morphine, essential medications for PC, were only available for 11% of serious health-related conditions.^1^

The WHO Public Health Model has identified that adequate policies, drug availability, training for health professionals and the public, and implementation at all societal levels are four important elements for integrating PC. Unfortunately, Honduras lacks these elements, hindering resource allocation, and the quality of life for patients and families affected by serious illnesses,^9^ which can ultimately lead to catastrophic expenditure.

A household incurs catastrophic expenses when its out-of-pocket payments for basic needs exceed 40% of its household capacity or current income.^10^ This is especially true in Honduras, where 51.7% out-of-pocket payment was reported in 2021.^11^ This results in a low population coverage rate, as poor households spend most of their income on basic needs such as food and housing and very little or nothing on health.^12^

With 74% of families living in poverty,^13^ many are unable to travel abroad to receive PC services. They are suddenly forced to rely on informal caregivers (who will be addressed from now on as non-health caregivers) who are not employed or compensated by the healthcare system^14^ and include a wide range of individuals such as relatives, friends, neighbors, community leaders, or even religious figures, who are now caring for these patients at home without knowing how to manage their pain or emotional needs.^15^ The challenge intensifies when people are uncomfortable with the idea of death,^16^ leaving the patients to endure their final moments alone in agony.^17^

As the global population ages and the prevalence of various noncommunicable diseases rises, educating the public about PC is critical.^18^ Despite this need, most PC-related articles are aimed at health professionals,^19^ and few global efforts have been made to educate the public about PC.^20–22^ Last Aid, from Germany, is one example of a new initiative for teaching the public about PC, covering topics such as end-of-life care, advance care planning and decision making, symptom management, and cultural elements of death and grief.^23^ This is seen as essential to fostering compassionate communities^24^ helping recognize the notion that “end-of-life care is everyone’s responsibility.”^25^ Since 2014, Last Aid courses have been introduced in various countries, such as Germany, Denmark, Norway, Australia, and Brazil, helping people gain practical knowledge and feel more comfortable discussing and managing end-of-life situations.^26^ Another impactful intervention is PalliActive Caregivers (Cuidadores PaliActivos in Spanish), which was piloted in Colombia. This program provided non-health caregivers with educational booklets and interactive applications to teach essential caregiving skills. Caregivers learned how to manage daily tasks such as feeding, bathing, symptom control, and even administering subcutaneous injections when necessary, empowering them with confidence in their caregiving abilities.^27^ In the United States, a qualitative study explored the impact of a soap-opera-style bilingual educational intervention called “Caregivers Like Me,” which was designed specifically for Latino caregivers, who have historically underutilized end-of-life resources. The intervention helped participants recognize the importance and normalcy of accepting help, prioritizing self-care, and utilizing the available resources.^28^

Implementing similar models in countries facing shortages of PC resources and socioeconomic challenges, such as Honduras, could help alleviate the burden on families^29^ who are not trained to care for loved ones. A PC training program has the potential to equip non-health caregivers with the skills and knowledge to provide effective and compassionate care at home. Thus, the study aimed to explore the feasibility of a PC training initiative for non-health caregivers in Honduras.

We defined and deconstructed the term “feasibility” as a comprehensive exploration of three main dimensions: participants’ experiences and perceptions of PC, the opportunities and challenges involved in implementing such a program, and if deemed feasible, recommendations on its design and implementation. Our understanding of feasibility encompasses not only the possibility but also the likelihood of successful implementation, acknowledging that it cannot be assured unless all these dimensions are thoroughly examined and addressed. Our study was exploratory in nature because evaluating feasibility in a context with limited prior research necessitated understanding the broader landscape of PC in Honduras.

## Methods

### Qualitative approach and research paradigm

The qualitative method employed in this study was latent content analysis. This method takes the manifest analysis to a hermeneutical interpretation, uncovering implicit meaning that is inferred rather than explicitly stated, exploring beneath the surface of the text.^30,31^ This approach allows the information to move from the specific to the general in a more abstract manner,^32^ resulting in themes grounded in the data.^31^

The chosen paradigm for this study is interpretivism, which emphasizes understanding how individuals perceive and interpret their surroundings.^33^ Interpretivism asserts that knowledge and meaning are not straightforward, objective truths but are instead socially constructed through interactions and influenced by the contexts in which they arise.^33^ This paradigm facilitates the exploration of latent content, allowing for a richer and deeper understanding of the topic.

### Researcher characteristics and reflexivity

The Principal Investigator (PI) is a Honduran national with extensive experience as a licensed nurse, project manager, and health supervisor for the Ministry of Health of Honduras. The PI’s background, including both clinical practice and personal experience as a caregiver, provided valuable insight into the context and dynamics of the research. This preunderstanding of the informants’ surroundings helped shape the interview guide^34^ and allowed the PI to identify appropriate participants who could meaningfully address the research’s main objective.

To maintain balance and objectivity, the PI actively engaged in reflexive practices, including regular peer debriefings and journaling impressions as a method of bracketing personal assumptions and biases.^31^ The interview guide was carefully designed with open-ended and probing questions to ensure that the study remained focused on participants’ voices and lived experiences, rather than assumptions drawn from the PI’s prior knowledge.^35^ Through these practices, the PI ensured that preunderstanding enhanced the study’s relevance without compromising its rigor, ultimately reaching a true hermeneutic interpretation.^36^

### Context

**Figure 1.**
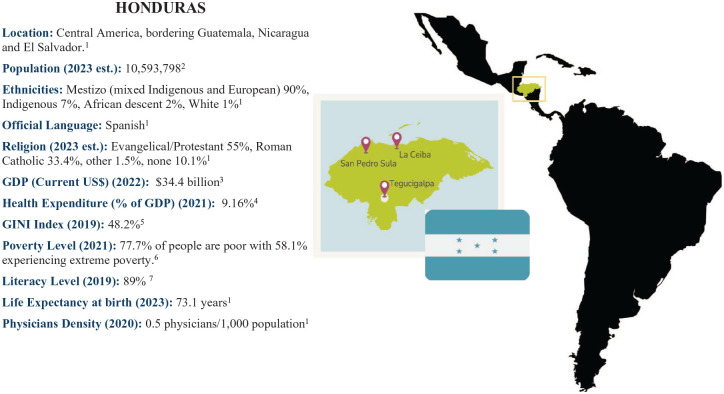
Context. Source: ^1^Central Intelligence Agency. The World Factbook: Honduras 2024. ^2^World Bank. Population, Total—Honduras 2023. ^3^World Bank. Gross Domestic Product (GDP) (Current US$)—Honduras 2023. ^4^World Bank. Current Health Expenditure (% of GDP)—Honduras 2021. ^5^World Bank. GINI index—Honduras 2019. ^6^Instituto Nacional de Estadística. LXXII Encuesta de Hogares para Medir Pobreza 2021. 2021. ^7^World Bank. Literacy rate, adult total (% of people ages 15 and above)—Honduras 2019.

The research was conducted in three major cities of Honduras: Tegucigalpa, the capital and largest city^37^; San Pedro Sula, the country’s main economic engine due to its robust industrial sector^38^; and La Ceiba, a city that drives cultural development through its vibrant tourism industry^39^ (Figure 1). PC specialists were interviewed in Tegucigalpa, San Pedro Sula, and La Ceiba. Decision makers were interviewed in La Ceiba and Tegucigalpa and both groups were identified using medical or government directories. The researcher conducted interviews with patients and non-health caregivers who attend the Fundación Pro Cancer,^40^ this research’s host institution, located in La Ceiba, all of whom were invited to participate through the health professionals in charge of their care.

### Sampling strategy

**Figure 2.**
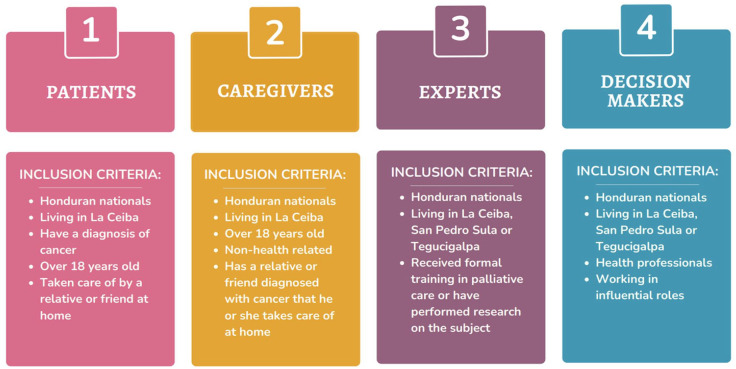
Participants’ inclusion criteria.

Maximum variation sampling was employed to gather a diverse range of participants to explore the feasibility of a program like this.^34^ This diversity captures the variations in the roles and functions of participants, which includes both facilitators and recipients of PC. This helps guide us through their perspectives from different lenses, while at the same time, allowing us to understand their challenges, and existing opportunities, as well as their needs, ultimately helping to answer the research question. Participant recruitment was based on carefully developed eligibility criteria (Figure 2) to ensure that the research was attainable and aligned with the research objectives. These participants fell into one of four categories: Patients, non-health caregivers, PC experts, and decision makers (Table 1).

**Table 1.** Key groups selection reasoning.

Key groups	Reasoning
Patients	Because they are the direct recipients of PC at home, it is critical to describe their experiences, needs for these services, as well as their expectations and perceptions of the utility and feasibility of a program of this type for non-health caregivers.
Caregivers	They provide a direct understanding of the practical and emotional needs of providing PC at home, as well as their perception of the utility and feasibility of a home PC training program aimed specifically at them.
PC experts	PC experts offer specialized knowledge on best practices in PC as well as the elements that must be included in a training program. They can also assist in identifying program development challenges and opportunities.
Decision makers	Health-related decision makers are critical in determining the level of interest and willingness to support and integrate the program in Honduras. They can also assist in identifying program development challenges and opportunities.

PC, palliative care.

A minimum of five participants from each key group were planned to be interviewed. After 20 interviews, 5 additional participants were interviewed to enrich the study. At this point, no new information emerged from the data, and it was considered that further data collection would not significantly contribute to the understanding of the studied phenomenon.

### Ethical issues pertaining to human subjects

Data that is unrelated to the research questions and reveals the participant’s identity was not collected, complying with the principle of nonmaleficence.^31,41^ Participants were presented with an informed consent form, which outlined the research’s purpose, privacy rights, and the option to decline answering questions or withdraw from the study at any time without explanation, ultimately complying with the autonomy principle.^31,41^ The study would not directly benefit the participant. Because this study deals with a sensitive subject such as PC among patients diagnosed with cancer, it is critical that the beneficence principle be followed. This was accomplished by keeping interviews brief and allowing participants to take appropriate breaks.^41,42^ The inclusion of four different groups of participants ensures that the research captures a variety of perspectives, thus promoting inclusion and the principle of justice and empowerment in the study.^41^ Since this study included human participants, ethical approval was requested and granted by the Biomedical Research Ethics Committee of the Faculty of Medical Sciences of Honduras (IRB: 0000-3070), adhering to the Declaration of Helsinki’s ethical guidelines.^43^

### Data collection methods

The study employed individual semi-structured interviews which is a flexible and interactive conversation method that contains closed and open questions which enables the conversation to broaden and touch on latent elements relevant to the research objective.^44^ Due to the differences between groups as a result of the chosen sampling technique, tailored interview guides for each group were developed based on the four categories to which participants were assigned^44^ (Supplemental Appendix 1). This triangulation of sources provides the opportunity to cross-verify responses, ensuring a more robust understanding.^31^ Data collection and analysis for this study took place over a 3-month period, beginning after receiving approval from the ethics committee.

### Data collection instruments and technologies

These interview guides were developed in a comprehensive manner to capture a complete picture of the feasibility of a PC training initiative in Honduras. By exploring various dimensions, such as participants’ experiences, logistical challenges, and specific training needs, the guides enabled a thorough assessment of factors that could impact the program’s implementation. The PI conducted all interviews, each lasting approximately 1 h. Interviews were recorded using a Sony ICD-PX470 audio recorder, with the recordings later transcribed for data analysis. Interviews with decision makers and PC experts were recorded in their workplaces, while non-health caregivers’ and patients’ interviews were recorded during their medical consultations at their care centers. Impressions relevant to the chosen collection method were recorded in a journal.

### Data processing

All interview recordings were transcribed verbatim by the PI, first in Spanish, the original language, and then translated into English. Since the PI is a Honduran national who has received bilingual education since primary school and has prior experience in conducting qualitative research, there is assurance that no meanings were lost in translation during the transcription process. Verbatim transcriptions of the interviews were written using Microsoft Word Document. To safeguard the privacy of the participants, the transcripts do not include their names, but instead use alphabetical-numeric codes for each one. Only the PI has access to the recorder and journal. At the end of this study, all the recordings were destroyed.

### Data analysis

**Figure 3.**
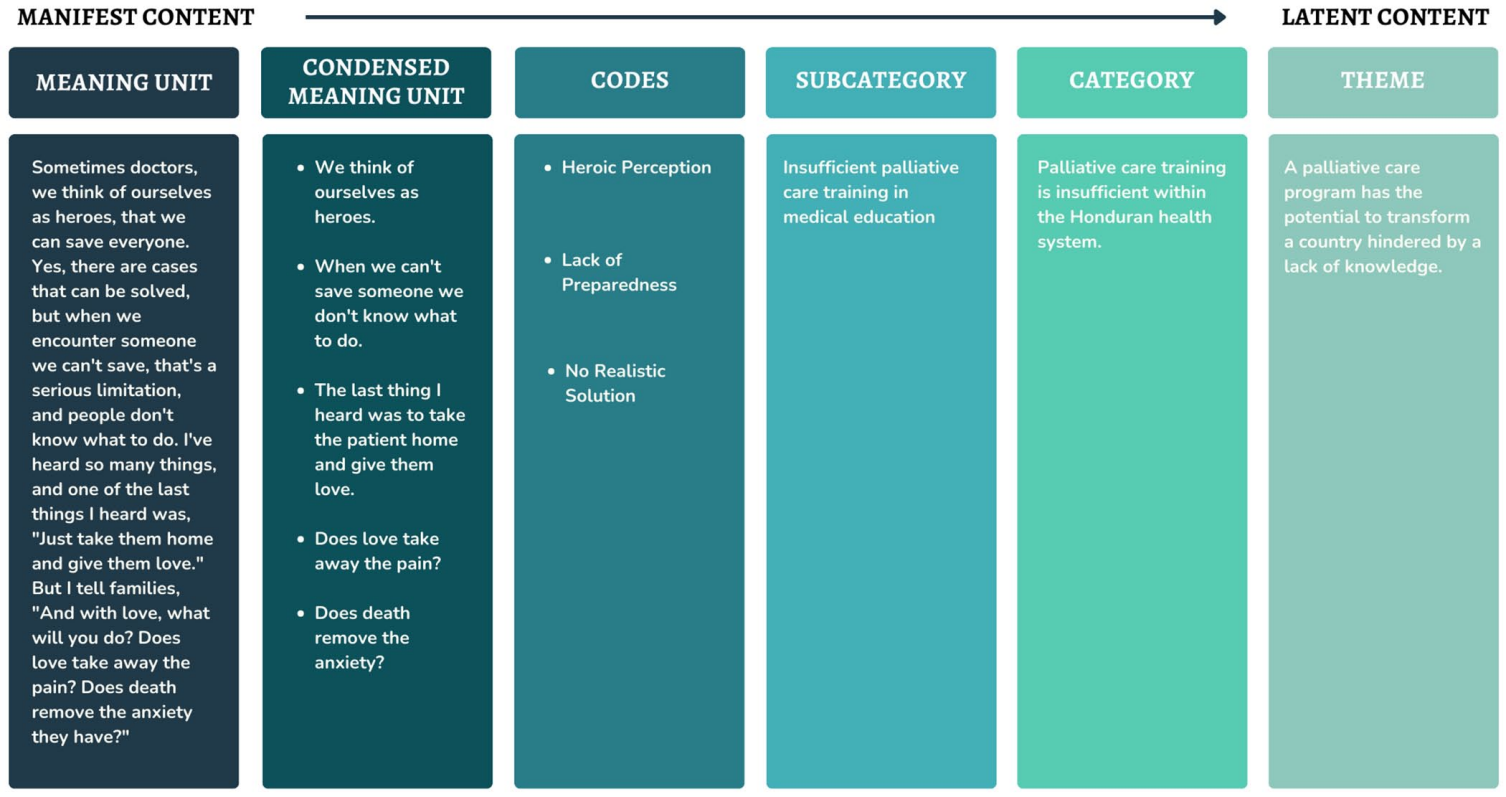
Sample of analytical process moving from manifest to latent content (left to right).

The transcribed text was subjected to a series of analytical steps progressing from a phenomenological interpretation to more hermeneutical meanings^45^ (Figure 3). This means that instead of merely taking in the words as they are spoken, the process also explored the emotions, intentions, and contexts behind what was said, allowing for a fuller understanding of why the interviewees chose those specific words. The data analysis was done by a single coder, the PI.

The first step was becoming acquainted with the text by reading it several times while keeping the research objective in mind. The PI’s preunderstanding of the informant’s surroundings aided to better interpret the collected data.^31,36^ Next, the transcripts were divided into smaller sections called “meaning units,” which represented different ideas or themes. To make analysis easier, these meaning units were condensed to focus on their main ideas.^31,36^ Then, short descriptive codes, no longer than five words, were created to summarize these condensed meaning units. This step helped organize the data and identify connections between different ideas.^46^

Following this, these codes were compared to each other and grouped into categories based on visible similarities and a straightforward analysis where the words were taken at face value (manifest content).^35^ However, since participants provided rich and detailed answers, a wide range of codes emerged. To manage this complexity, similar codes were grouped into subcategories.^46^ The subcategories were then analyzed and grouped together based on their connections to form a category. Finally, these categories were grouped, and themes were developed to capture deeper meanings behind the data and achieve a higher level of abstraction (latent content),^35^ to tie all data together in a metaphorical manner.^47^ Unlike a quantitative study, this process required constant back-and-forth reflection and refinement.^35^

### Techniques to enhance trustworthiness

The triangulation of data sources allows for the comparison of perspectives and knowledge across the four key groups, providing a holistic understanding of the topic while ensuring its credibility.^31,48^ Peer debriefings were conducted after developing the categories and themes to help evaluate the PI’s role in the research process, resolve inconsistencies, and further ensure the study’s credibility.^31^ Throughout the data processing and analysis process, the PI kept a journal of her impressions and decisions, which she only had access to. This will allow for a more detailed audit at the conclusion of the investigation, in accordance with the dependability and confirmability criterion.^49^ The study did not attempt to obtain a statistical generalization, but rather an analytical generalization of depth of thought, using a sample that provides comprehensive results. These findings can provide knowledge that can be applied to similar situations outside of the study group. This knowledge is not limited by demographics but focuses on understanding and solving problems in larger contexts, meeting the transferability criterion.^31^

The reporting of this study conforms to the Standards for Reporting Qualitative Research (SRQR) guideline^50^ (Supplemental File 1).

## Findings

In this study, a total of 25 participants were interviewed. These included five patients, six non-health caregivers, eight experts, and six decision makers. Out of the 25 participants, 14 were women and 11 were men (Table 2).

**Table 2.** Interviewees’ background information.

Key group	Participant	Lives in
Patients	P1	La Ceiba
	P2	Tela
	P3	La Ceiba
	P4	La Ceiba
	P5	La Ceiba
Caregivers	C1	La Ceiba
	C2	La Ceiba
	C3	La Ceiba
	C4	Tela
	C5	Guanaja
	C6	La Ceiba
PC experts	E1	Tegucigalpa
	E2	Tegucigalpa
	E3	Tegucigalpa
	E4	Tegucigalpa
	E5	La Ceiba
	E6	La Ceiba
	E7	San Pedro Sula
	E8	Tegucigalpa
Decision makers	D1	Tegucigalpa
	D2	Tegucigalpa
	D3	Tegucigalpa
	D4	La Ceiba
	D5	La Ceiba
	D6	Tegucigalpa

PC, palliative care.

The manifest content analysis generated 299 codes, 26 subcategories, and 10 categories. The latent content analysis generated three overarching themes (Table 3).

**Table 3.** Table of results.

Subcategories	Categories	Theme 1
Patient’s initial response to PC	PC is tumultuous and emotional.	The tumultuous reality of navigating PC in a low- and middle-income country.
Patient’s journey throughout PC		
Caregiver’s initial response to PC		
Caregiver’s journey throughout PC		
The impact of PC on family dynamics		
Patients and caregivers’ experiences seeking healthcare	PC in Honduras is made difficult by the challenges of poverty.	
Experts’ experiences providing PC		
Current realities of Honduran households		
Finding meaning during difficult situations		
Subcategories	Categories	Theme 2
Insufficient PC training in medical education	PC training is insufficient within the Honduran health system.	A PC program has the potential to transform a country hindered by a lack of education.
Population’s misconceptions	There are misconceptions about PC in the Honduran population.	
Lack of political will	PC is underrepresented due to governance gaps.	
Systemic issues across the country		
Corruption as a persistent adversary		
PC’s diverse range of benefits	Non-health caregivers demand a PC training program.	
Progress made in PC development	PC in Honduras is emerging.	
Honduras is a country with potential		
Active support among diverse stakeholders		
Subcategories	Categories	Theme 3
Caregivers’ needs and rights to effective and compassionate PC	The program must meet people at their point of need.	A sustainable and feasible PC program is built from a bottom-up approach.
Necessary knowledge and skills for caregivers		
Delivery and styles of learning		
Requires an interdisciplinary team of health professionals	Sustainability depends on intersectoral action.	
Support from different sectors		
Caregivers can take many forms		
Principles that guide PC	It requires a framework that adheres to best practices.	
Comprehensive program design, execution, and evaluation		

PC, palliative care.

### Theme 1: The tumultuous reality of navigating PC in a low- and middle-income country

#### PC is tumultuous and emotional

Patients’ initial response to PC involves an assortment of emotions, such as shame, fear, denial, resignation, anxiety, frustration, self-isolation, depression, and resentment. Patients often experience suffering as their illness progresses, as well as low self-esteem, feeling like a burden to the family, losing their independence, being overlooked, and changes in their lifestyle. Moreover, patients had to frequently alternate between the hospital and their homes, and they expressed that being in the hospital is equivalent to being locked up.

Caregivers reported a range of emotions after learning the patients’ diagnoses and their new and unexpected role as non-health caregivers. This included feelings of worry, confusion, uncertainty, and sadness.

[. . .] it becomes a moment where you feel useless for not being able to do anything [. . .] I would have liked to receive psychological help to be prepared for everything that was coming because one always hopes that the person will recover [. . .]. (C2)

Non-health caregivers expressed that they have had to be resourceful with what they rely on. They have experienced doubts, discomfort, exhaustion, overwhelm, and exclusion from healthcare professionals. They have also had feelings of regret about how they have provided care, felt misunderstood, and neglected their mental health during the process. They have made personal sacrifices, helped out of love, and shown strength even when they did not have it.

[. . .] in the hospital they know what they have to do, they know what the best care is, because I do what I can here, but I don’t have the knowledge like a doctor or a nurse, that’s the difference. (C6)

Participants described how, during the PC process, family members often become absent or inconsiderate, and caregiving tasks become the responsibility of only one relative.

Last year, I think, in August, [. . .] they operated on my small intestine, and thank God I’ve recovered well. Lately, I feel like there’s a lump in my belly button, and I asked the doctor [. . .] and she says it’s some strain I did and I got a hernia because, [. . .] I’m alone, [. . .] my sister was supposed to take care of me, but I would yell at her, and she never listened, so I had to learn to see how I could get up on my own [. . .] it’s important to have someone to rely on, because that day when I needed to get up and my sister was out there playing dominoes. . . she never came. From that moment on, I said I wasn’t going to depend on anyone. [. . .] there are some family members who. . . how can I say it? don’t even care about you. I think my family sometimes expects me to take care of them. (P5)

#### PC in Honduras is made difficult by the challenges of poverty

All participants expressed discontentment with current health services, as evidenced by their experiences with unaffordable healthcare services, a lack of healthcare institutions in their communities, inadequate facilities, poor management, apathic health professionals, and mistreatment. Ultimately, most participants expressed distrust in the public health system.

[. . .] that’s what we see nowadays—a lack of hospitals, shortages of supplies, damaged equipment. (D5)

All participants reported that the current reality in Honduran households is characterized by family members who have migrated or moved away, high rates of comorbidity, financial hardship, low levels of education, and limited employment opportunities.

Patients and caregivers both expressed that during these difficult times, they tend to rely on faith, take breaks from the situation by engaging in other activities, and seek advice from others who have been through similar experiences.

### Theme 2: A PC program has the potential to transform a country hindered by a lack of education

#### PC training is insufficient within the Honduran health system

Both decision makers and experts expressed dissatisfaction with the country’s current medical education regarding PC. They reported that the current syllabus is out of date and heavily focused on treatment rather than prevention. Participants indicated that health professionals do not know how to manage a patient in PC, which causes them to behave insensitively, play the role of hero with these patients, and provide unrealistic solutions to non-health caregivers.

Well, you know, if there’s no knowledge, there’s no teaching, right? In undergraduate training [. . .] it’s not very well-known, which leads to the fact that if you don’t know something, well, you don’t know how to deal with it, how to help, how to improve. [. . .] it becomes an area that’s basically abandoned and never treated as a priority. That’s when we start facing a lot of limitations [. . .] like managing pain or meeting patients’ needs [. . .]. (E5)

#### There are misconceptions about PC in the Honduran population

All groups reported that misinformation about PC is prevalent in the country. Some participants stated that PC is associated with death and that it is only for elderly patients or those with cancer. Furthermore, other participants acknowledged that this was a taboo, controversial, and even utopian topic.

[. . .] one thinks that it is related to a terminal stage. [. . .] When people talked about PC, that was my first impression, but later I realized that it’s not just that; it’s all that comes with it and the treatment [. . .] The first impression I had was of someone in bed sedated; from time to time, uh, feed him or whatever and again leave him sedated in bed to avoid pain and just wait for God to decide [. . .]. (C1)

#### PC is underrepresented due to governance gaps

Experts and decision makers stated that Honduras lacks a PC policy to help guide health institutions and professionals in their daily work. This was evident from the lack of budget and interest shown by decision makers in this topic as reported by them. In addition, they claimed that initiatives in Honduras are primarily motivated by political agendas, depending on which party is currently in power or whether the initiative will help them improve their public image.

Participants stated that multiple issues are affecting the country at the same time, including a scarcity of PC experts, brain drain, and unequal distribution of experts across the country.

[. . .] you can’t say that it’s one of the priority careers that young doctors in Honduras are seeking. If you come as a PC specialist, the State doesn’t hire you because [. . .] it’s not in the country’s health human resources framework [. . .] they hire you as a general practitioner, but not as a PC specialist. It’s a mess, and you’d have to make those kinds of reforms first. (D2)

Every participant discussed how corruption affects their surroundings. Experts and decision makers described how things operated in their workplaces, witnessing a lot of nepotism and a lack of accountability. Patients and non-health caregivers described how this affects access to healthcare, including distrust of health institutions, because of resource misappropriation.

#### Non-health caregivers demand a PC training program

All participants agreed that a PC training program can provide numerous benefits, ranging from providing relief to people, making connections, and improving patients’ quality of life, to broader benefits such as disseminating knowledge, improving healthcare provision, and lowering healthcare costs.

[. . .] it would empower a non-health caregiver [. . .] because [. . .] the system doesn’t reach the furthest communities of Honduras. (E1)

[. . .] It is very important to learn; you never know the twists and turns of life. Just think about it, I never imagined I’d be in that situation with my mother and now my father. (C1)

#### PC in Honduras is emerging

Experts and decision makers stated awareness about efforts that have been put forward regarding PC, such as the existence of a PC institution, PC guidelines developed by institutions, PC units already established in hospitals, and recently implemented PC courses targeted to health professionals.

A manual was implemented for parents and caregivers of patients in PC. When a parent comes to the outpatient clinic, they are given this little manual that shows the different symptoms the patient may experience and how the family member should respond. This is primarily so they have a tool at home on what to do if the patient vomits, experiences pain or has a fever. In a way, we are educating the patient through this manual [. . .] what we’ve achieved is that families understand their loved one’s symptoms a bit better. How have we noticed this? Many times, families used to come to the hospital or call us saying the patient was in pain or had a fever. . . they didn’t know what to do. The answers to those questions are in the manual. . . this has reduced anxiety in the family and calls to the hospital. (E8)

All participants identified several opportunities for implementation. In the medical field, PC is already well-known in oncology, experts have specialized expertise due to their specializations in other countries, and health professionals never pass up an opportunity to learn something new. Outside of the medical field, people are not entirely unaware of the concept of PC; however, due to a lack of formal training in the subject, it is frequently associated with misconceptions. There is often someone within our circles who has encountered it in some capacity. These firsthand experiences offer valuable insights into what PC involves, even without formal education in the field. Moreover, communities tend to be tightly knit, offering various forms of support to their members.

Hondurans really have the characteristic of helping each other. [. . .] I have a case of a girl who learned how to feed through a gastrostomy because she saw a family member doing the same for a patient with cancer, and currently, her family member had a gastrostomy placed and now she knows how to feed her family member. This has the ability to help other people in the community as well. (E1)

Experts and decision makers discussed some well-known contributions made by various sectors. These were faith-based organizations, universities, health professionals, experts, and politicians who shared common interests and wanted to see PC integrated and developed in Honduras.

### Theme 3: A sustainable and feasible PC program is built from a bottom-up approach

#### The program must meet people at their point of need

Patients expressed a desire to die peacefully at home with their families present. They also expressed a desire for emotional, social, and spiritual support, independence, comfortability, motivation, and the right to be informed. Non-health caregivers expressed their needs, which included learning how to provide care, hospital access for complex procedures, companionship, financial support, access to equipment such as wheelchairs, and preparing for death and the closure that follows.

[. . .] for example, in my case, if someone had advised me about this, we wouldn’t have made several mistakes. So, I do believe that educating people about PC is very important. (C2)

All participants stated that non-health caregivers should learn a diverse range of skills and topics related to nutrition, hygiene, injection administration, vital sign monitoring, pressure ulcer prevention, wound care, feeding, learning about illness, and reading laboratory test results, as well as how medications work, their side effects, and dosage. Furthermore, aspects involving emotional intelligence were constantly brought up, such as how to talk, how to de-escalate a conflict, and how to self-regulate emotions.

If we have a person with cancer, it should be understood that the family also has the cancer problem, right? Because it’s not only the person who is suffering, but their entire environment. Here, we only really see the biological, physiological suffering of the person, and they only care about the one with the health problem and not about the caregiver, who may also experience burnout. It could be, for example, an elderly person taking care of another elderly person with cancer, or it could be a teenager taking care of their mom or dad because the other person in the family has to work. This young person may not have the tools to cope with these types of illnesses [. . .] This shouldn’t be solely focused on clinical care; emotional care for the patient and their caregivers should be integrated in a way that particularly addresses facing death and the stages it entails, because it’s a painful process. (D3)

Participants expressed the need for a training program that accommodates non-health caregivers’ diverse learning styles, including kinesthetic, visual, and auditory, as well as individual and group learning preferences. They also stated that easy language should be a must, along with reinforcement and different levels of difficulty based on prior knowledge or experience in caregiving. They also expressed a need for a variety of delivery options, tailored to their circumstances, whether online or in person, at a suitable location for them.

Attending an in-person class and putting what I read into practice. Learning by doing, experiencing it firsthand, that’s the best way. I think it would be best, just like in the case of people going through these kinds of processes or who have gone through them, if they could share their experiences with others to gain some knowledge that will help them in the future. (C3)

#### Sustainability depends on intersectoral action

Experts and decision makers expressed the importance of implementing a PC training program aimed at non-health caregivers through a collaborative approach, which includes a multidisciplinary team of health professionals, as well as the various types of caregivers who exist and are willing to take on the role, and the support of various sectors such as government, community, universities, patient unions, churches, among others.

This is an ongoing, comprehensive job, right? It’s not just confined within the walls of a hospital. It’s not just about addressing healthcare professionals; it also has to include family members and support networks. (E4)

#### It requires a framework that adheres to best practices

According to expert responses, PC should be of high quality, begin at the time of diagnosis, and consider people’s realities. This requires deep and ongoing commitment, open communication, and ethics in decision making that can ultimately help dying well a reality.

[. . .] We should think about dying well, closing life with dignity. We are all going to have a closure in life. . . all of us. So, if we are going to strive to improve the quality of life, why not also strive for a good quality of death? (E4)

Decision makers and experts agreed that the design and implementation of a PC training program should include pilot tests and periodic assessments before, during, and after execution.

[. . .] In some indigenous communities, there are variations in how they feel and think about death. So, we would need to make some adaptations based on qualitative research, for example, on how this process is coped with [. . .]. (D6)

## Discussion

The study’s approach, of exploring both the emotional and practical needs of non-health caregivers, the socioeconomic barriers they face and opportunities they count on, as well as their perspectives on the delivery and content of a potential program, evidenced the feasibility and importance of a such initiative as well as its replicability in low- and middle-income countries with similar challenges.

It highlights how becoming a PC patient or non-health caregiver can be emotionally draining.^51^ It is unfamiliar territory for both parties, as patients are dealing with serious health suffering and non-health caregivers are navigating the complexities of caring for someone and potentially losing them. This emotional burden can cause a strain in family dynamics,^51^ ultimately leading to an unequal distribution of responsibilities, with only one person willing to endure the entire process. These sole non-health caregivers are then forced to adapt their day around the patient’s everyday routine since they can’t afford additional help.^52,53^

Providing optimal care at home can become challenging for non-health caregivers when they lack confidence in essential tasks such as feeding patients, proper lifting techniques, wound care, administering medications correctly, and understanding the progression of the illness. Furthermore, the inaccessibility of health services, the lack of information, and guidance provided by health professionals,^54^ as well as the financial hardships at home, further contribute to the emotional rollercoaster.^53^ This creates a silent conspiracy between patients and non-health caregivers where none dares to address their true thoughts to each other. This causes and exacerbates feelings of abandonment and loss of independence^53^ in the patient, while anxiety and doubt plague the non-health caregiver. After the patient dies, the non-health caregiver’s previous feelings are replaced by new ones, such as regret and unresolved grief. The only thing left for them in these situations is to find meaning or relief in small things, to avoid succumbing to the harsh reality they face. However, this often only provides fleeting joy. Other studies conducted worldwide including in China,^55^ The Netherlands,^56^ Chile,^57^ and Colombia,^58^ have found similar results, demonstrating that, despite differing contexts, patients and non-health caregivers all fear and want the same, effective support and compassionate care.

The harsh experiences of individuals navigating PC stem from the long-standing inequality that has marked Latin America throughout history, as highlighted in much of the literature.^59–61^ However, the responses of participants led to the conclusion that these issues should not be considered “inequality,” but rather “*inequity*” since it touches upon moral and ethical dimensions referring to situations that are unnecessary, avoidable, but also regarded as unfair and unjust.^62^

Inequity occurs when people have little or no control over external factors, as demonstrated by the scenarios of patients and non-health caregivers. A sense of injustice grows when challenges accumulate and are reinforced, predisposing people to poor health.^62^ And surprisingly enough, this issue is not confined to low-resource settings. Similar disparities have been noted in more affluent countries such as Denmark, where, even with a universal healthcare system that guarantees equal rights, PC is unevenly allocated along socioeconomic lines, with wealthier patients receiving more focused attention and care.^63^

In Honduras, inequity stems from a lack of education. The insufficient integration of PC in medical education^64^ leaves non-health caregivers to provide empirical care at home, fostering misconceptions and reducing public pressure for supportive services. As a result, the decision maker’s commitment to PC decreases,^64^ opening the opportunity for corruption to grow. A lack of education, if not addressed, represents a self-perpetuating cycle^65^ in which a lack of education leads to more lack of education.^66^ These findings align with similar conclusions drawn in other studies in other countries where participants voiced these aspects as recurrent obstacles of PC integration and development in low- and middle-income countries.^67,68^

While Honduras faces the typical obstacles of lower-middle-income countries, it is important to acknowledge the country’s gradual progress in developing and integrating PC, proving its potential for innovative health initiatives, such as a training program aimed at non-health caregivers. Numerous organizations such as Fundación Pro Cáncer,^40^ Centro de Cuidados Paliativos Asociación OMEGA,^69^ Fundación Hondureña para el Nino con Cáncer,^70^ Hospital María Especialidades Pediátricas,^71^ Hospital Escuela Universitario,^72,73^ and the Universidad Nacional Autónoma de Honduras^74^ among others, have been key in advocating for these efforts in recognition of the value and benefits of PC. Their initiatives aim to dispel misconceptions, raise awareness about PC, and strengthen efforts to train non-health caregivers.

The fact that most people have had some experience caring for someone at home enhances the feasibility of implementing a PC program in Honduras, as even non-health caregivers with low education levels, can relate to PC through their personal experiences as caregivers or observers. Furthermore, Honduran communities are united, as evidenced by the diverse range of non-health caregivers who fill this role,^75^ including neighbors, friends, and family members. This can enable the implementation of training initiatives with the support and monitoring of a community that is concerned not only with its own well-being but also with that of others. This illustrates how individuals and communities unite during challenging times to collaborate and find solutions together, because as stated in other studies, “end-of-life care is everyone’s responsibility.”^25,76^

**Figure 4.**
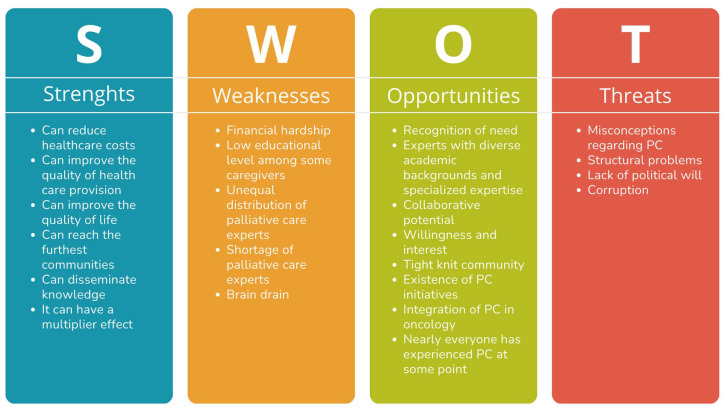
Strenghts, Weaknesses, Opportunities and Threats (SWOT) analysis.

Implementing a PC training program could offer numerous benefits. It would empower non-health caregivers without compromising their mental health or leading to burnout, while also enhancing the quality of life for patients at home.^77,78^ In a country where 74% of families live in poverty,^13^ such programs could provide a safe haven by reducing the costs of frequent trips^67^ and eliminating the need for repeated work sick leaves^52^ or job resignations. It would also foster a community where everyone is comfortable with the concept of death and collectively contribute to the care of others, creating a multiplier effect. In addition, it could benefit the healthcare system by reducing unnecessary hospital admissions and costs^67^ (Figure 4).

To implement such a program and ensure its feasibility and sustainability, it must be designed using a bottom-up approach,^79^ as it is necessary to understand people’s realities and the challenges they face, enabling more effective and relevant interventions. To achieve this, it is critical that implementers actively contribute to policymaking rather than simply receiving and following orders sent from above.^79^

**Figure 5.**
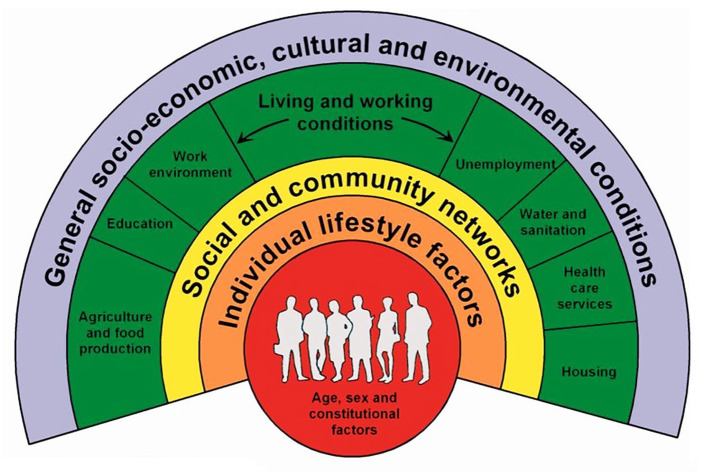
Dahlgren and Whitehead model. Source: Dahlgren and Whitehead, 1991.^82^

While our focus is on Honduras, it is important to acknowledge the existing differences within this population.^80^ To better understand these disparities or inequities, we can refer to the Dahlgren-Whitehead model, which illustrates the intricate relationship between an individual, their environment, and their health^81^ (Figure 5). This framework is particularly valuable in this study as it illustrates the interplay of various social determinants that affect both patients and non-health caregivers. Understanding these interconnected factors is essential for developing and executing effective initiatives such as a PC training program.

**Figure 6.**
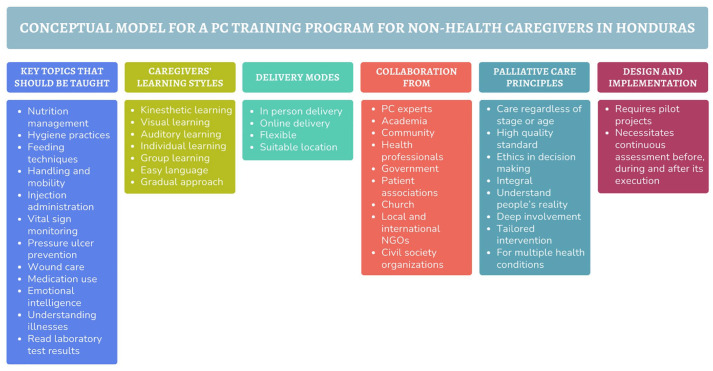
Conceptual model of how a palliative care training program for non-health caregivers in Honduras should be expressed by participants.

Too often, however, initiatives are designed from a unidirectional perspective,^64^ failing to move beyond the assumption that the health sector alone is responsible for driving change.^81^ Such a limited view overlooks the broader context in which health determinants operate^81^ and undermines the need for multi-sectoral collaboration. In failing to address these interconnections, programs risk falling short before they even begin. In Latin America, where inequities affect nearly every aspect of life, an intersectoral approach is not just ideal but essential.^59^ For a PC training program to have a lasting impact, it must integrate insights from various sectors and be attuned to the unique circumstances of each community, effectively supporting the 2030 Agenda pledge to “leave no one behind.”^83^ To do so, we need to understand the gaps in skills and knowledge among non-health caregivers, how they learn best, how to adapt to their living circumstances, the support systems available to them, their relationship with the patient, the time they have, and their current level of education (Figure 6). Without a thorough understanding of the latter, even the most successful PC programs from more developed countries are likely to fail upon implementation.

Through the research findings, it became evident that a PC training program could significantly alleviate the caregiving burden in Honduras where PC services are scarce, equipping non-health caregivers with essential skills and confidence to provide effective support at home. This research contributes to a deeper understanding of how PC can be decentralized and community-supported, expanding the traditional healthcare model to include trained, non-health caregivers, and offering a sustainable, cost-effective model adaptable to various cultural and economic contexts. As countries with similar settings look to improve end-of-life care, this study provides actionable insights on implementing and scaling PC initiatives that prioritize compassionate, equitable support for patients, and non-health caregivers alike.

### Limitations: Trustworthiness and limitations of findings

One limitation of our study is that we were unable to interview participant groups across all three target locations: La Ceiba, San Pedro Sula, and Tegucigalpa. The identification and participation of experts and decision makers were limited by their locations at the time of data collection and by restricted access to up-to-date, functional government, and medical directories. Identifying patients and non-health caregivers depended on the availability and support of healthcare institutions, which made it more practical to rely on the research’s host institution.

While large sample sizes are unusual in qualitative studies, prioritizing depth over precision, the 25-sample size in this study is seen as a strength rather than a limitation. The size offers valuable insights into the research landscape of PC in Honduras, as it is the first study of its kind in the country. However, further research is needed to delve deeper into the topic and strengthen the evidence base.

Furthermore, while the topic guide may appear extensive, it was deliberately designed to ensure that no aspect of feasibility was overlooked. Each section was integral to forming a holistic understanding of the feasibility of a PC training initiative for non-health caregivers in Honduras. While this approach may have generated a broad dataset, each component contributed valuable insights, enhancing the depth and relevance of our findings to the study’s core objective.

## Conclusion

The study sought to explore the feasibility of implementing a PC training program for non-health caregivers in Honduras. Despite the obstacles inherent in a lower-middle-income country, the findings reveal that such a program is feasible and highly replicable in regions facing PC shortages.

A PC training program could provide diverse benefits at both the individual and country levels, by building on the advances done by multiple sectors that share the same values and goals. The impact can be accomplished through a bottom-up approach that considers the entire non-health caregivers’ context to provide accurate and relevant support that meets their physical and emotional needs.

As the first study of its kind in Honduras, more research is needed and should concentrate on enhancing these interventions and advocating for policy modifications to guarantee fair availability of PC. Efforts to achieve health equity should always be encouraged so information turns to actions capable of improving the health of a population that is tired of being left behind.

## Supplemental Material

sj-docx-1-pcr-10.1177_26323524251316897 – Supplemental material for “Don’t leave us behind”: a qualitative study exploring the feasibility of a palliative care training program for non-health caregivers in Honduras Supplemental material, sj-docx-1-pcr-10.1177_26323524251316897 for “Don’t leave us behind”: a qualitative study exploring the feasibility of a palliative care training program for non-health caregivers in Honduras by Sheryl Ruiz, Martin Stafström, Leda Parham and Luis Orellana in Palliative Care and Social Practice

## Declarations

**Ethics approval and consent to participate:** The study adhered to the Helsinki Declaration, which outlines ethical principles for research involving human participants. Ethical approval was granted by the Biomedical Research Ethics Committee of the Faculty of Medical Sciences of Honduras. Reference number is IRB: 0000-3070. Written informed consent was obtained from each participant.

**Consent for publication:** Not applicable.

**Availability of data and materials:** The data is not publicly available due to privacy and ethical restrictions.
