# Supplementary material for: “Don’t leave us behind”: a qualitative study exploring the feasibility of a palliative care training program for non-health caregivers in Honduras
Source: Palliat Care Soc Pract. 2025 Feb 6;19:26323524251316897. doi: 10.1177/26323524251316897 (PMC11803733; doi:10.1177/26323524251316897)
Supplement: sj-docx-1-pcr-10.1177_26323524251316897 – Supplemental material for “Don’t leave us behind”: a qualitative study exploring the feasibility of a palliative care training program for non-health caregivers in Honduras [file sj-docx-1-pcr-10.1177_26323524251316897.docx]

**Appendix 1.** Tailored interview guides and informed consents for key group.

**Research Study:** Explore the Feasibility of Implementing a Palliative Care Training Program for Non-Health Caregivers in Honduras

**Informed Consent for Patients**

You are cordially invited to take part in an interview for this palliative care study. However, before you decide to participate, you should carefully read this form to understand the purpose of the study.

The goal of this study is to gather information about your experiences and needs regarding palliative care services, as well as your expectations and perceptions about the usefulness of a palliative care training program aimed at non-caregivers, through a single interview. The study's findings may be useful for future decision-making in improving palliative care services in Honduras.

Your participation in this research will be entirely voluntary. If you agree to take part, you will sign the informed consent form and be given a copy. The researcher will interview you and record your responses with an audio recorder, which she will later transcribe for the study's analysis in a diary to which only the researcher will have access. Depending on your comfort level, the interview may last up to an hour and a half and will be carried out in person, at the Pro Cancer Foundation, which will be attended by the treating health personnel. You may take breaks as needed during the interview.

PATIENTS

Certain questions about the subject of study may be personal in nature and cause discomfort. If this occurs, you have the right to withdraw from the research at any time, if you choose, or to refuse to answer questions. Data unrelated to the research questions will not be gathered.

Do you agree to take part in the research?

I have read and understand the information on this form and agree to take part in this research.

**Full Name of Participant:__________________ Date: __________________ (DD/MM/YYYY)
Signature: _________________________________ Fingerprint (if necessary): ________________
Witness Name (if necessary): ___________________ Fingerprint (if necessary): ________________**


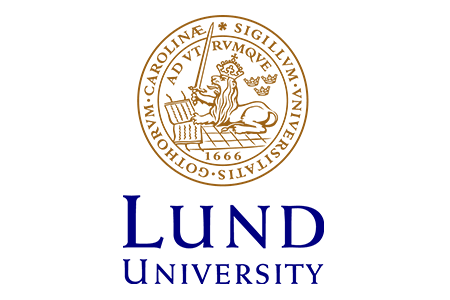
**
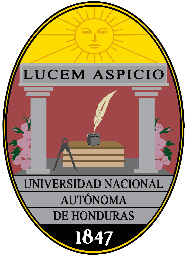
Researcher:** Sheryl Nicole Ruiz
**Public Health Master Student at Lund University | Bachelor of Science in Nursing**
**Cell phone:** (+46) 7######## **Email:** [s#######@student.lu.se](mailto:s)

**Research Study:** Explore the Feasibility of Implementing a Palliative Care Training Program for Non-Health Caregivers In Honduras

**Guide for a Semi-Structured Interview Guide for Patients**

**Experiences:**

1. How would you describe the palliative care you've received for your illness so far?
2. How does been taken care of at home by that person make you feel?

**Opinions:**

1. Do you believe that educating non-health caregivers about palliative care is important or useful? Explain
2. Based on your experience receiving palliative care, what do you believe a non-health caregiver should learn? Explain.
3. Do you believe that if any of your family members were given the opportunity to participate in a palliative care training program, they would agree to do it? Explain.

PATIENTS

**Feelings:**

1. What are the most pressing physical needs you have encountered while receiving palliative home care?
2. What are the most significant emotional or spiritual needs you have encountered while receiving palliative home care?
3. If you encountered difficulties or problems, how did you overcome them?

**Research Study:** Explore the Feasibility of Implementing a Palliative Care Training Program for Non-Health Caregivers in Honduras

**Informed Consent for Non-Health Caregivers**

You are cordially invited to take part in an interview for this palliative care study. However, before you decide to participate, you should carefully read this form to understand the purpose of the study.

The goal of this research is to gather information about your practical and emotional needs for providing palliative care at home, as well as your perception of the usefulness of a home palliative care training program for you, through a single interview. The study's findings may be useful for future decision-making in improving palliative care services in Honduras.

NON-HEALTH CAREGIVERS

Your participation in this research will be entirely voluntary. If you agree to take part, you will sign the informed consent form and be given a copy. The researcher will interview you and record your responses with an audio recorder, which she will later transcribe for the study's analysis in a diary to which only the researcher will have access. Depending on your comfort level, the interview may last up to an hour and a half and will be carried out in person, at the Pro Cancer Foundation, which will be attended by the treating health personnel. You may take breaks as needed during the interview.

Certain questions about the subject of study may be personal in nature and cause discomfort. If this occurs, you have the right to withdraw from the research at any time, if you choose, or to refuse to answer questions. Data unrelated to the research questions will not be gathered.

Do you agree to take part in the research?

I have read and understand the information on this form and agree to take part in this research.

**Full Name of Participant:__________________ Date: __________________ (DD/MM/YYYY)
Signature: _________________________________ Fingerprint (if necessary): ________________
Witness Name (if necessary): ___________________ Fingerprint (if necessary): ________________**


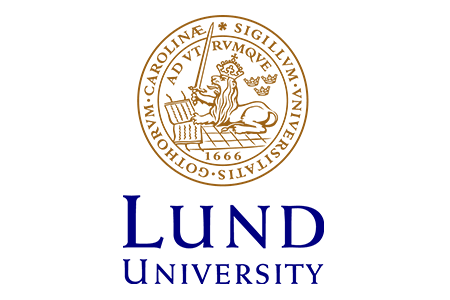
**
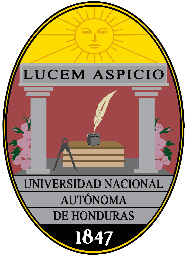
Researcher:** Sheryl Nicole Ruiz
**Public Health Master Student at Lund University | Bachelor of Science in Nursing**
**Cell phone:** (+46) 7######## **Email:** [s#######@student.lu.se](mailto:s)

**Research Study:** Explore the Feasibility of Implementing a Palliative Care Training Program for Non-Health Caregivers in Honduras

**Guide for a Semi-Structured Interview Guide for Non-Health Caregivers**

**Experiences:**

1. How would you describe the palliative care you have provided?
2. How does taking care of that person make you feel?

**Opinions:**

1. Do you believe it is important or useful to educate non-health caregivers about palliative care? Explain.

NON-HEALTH CAREGIVERS

1. What kind of things, based on your experience providing palliative care, would you like or need to learn? Explain.
2. What method do you believe you learn best? Reading books, attending personalized in-person classes, attending group classes, putting what you've learned into practice (doing), etc.? Explain
3. What are the challenges you see in participating in a palliative care training program?
4. Do you believe, at this time, you would agree to participate in a palliative care training program, if given the opportunity? Explain.

**Feelings:**

1. What are the most pressing physical, emotional or spiritual needs you've encountered while providing palliative care at home?
2. What challenges have you faced while providing palliative care to your caregiver? Explain
3. If you encountered difficulties or problems, how did you overcome them?
4. If you have previously discussed death-related issues with a friend or family member you care for, how did you do so? Do you believe it was done correctly?

**Research Study:** Explore the Feasibility of Implementing a Palliative Care Training Program for Non-Health Caregivers in Honduras

**Informed Consent for Palliative Care Experts**

You are cordially invited to take part in an interview for this palliative care study. However, before you decide to participate, you should carefully read this form to understand the purpose of the study.

The goal of this study is to gather information on specialized knowledge about best practices in palliative care and the necessary aspects to include in a potential training program, as well as the challenges and opportunities associated with program development, through a single interview. The study's findings may be useful for future decision-making in improving palliative care services in Honduras.

Your participation in this research will be entirely voluntary. If you agree to take part, you will sign the informed consent form and be given a copy. The researcher will interview you and record your responses with an audio recorder, which she will later transcribe for the study's analysis in a diary to which only the researcher will have access. The interview may last up to an hour and a half, depending on your comfort level, and will take place in person at a location of your choice. You may take breaks as needed during the interview.

PALLIATIVE CARE EXPERTS

Certain questions about the subject of study may be personal in nature and cause discomfort. If this occurs, you have the right to withdraw from the research at any time, if you choose, or to refuse to answer questions. Data unrelated to the research questions will not be gathered.

Do you agree to take part in the research?

I have read and understand the information on this form and agree to take part in this research.

**Full Name of Participant:__________________ Date: __________________ (DD/MM/YYYY)****Signature: _________________________________ Fingerprint (if necessary): ________________**


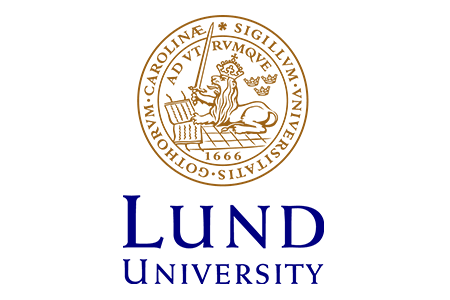
**
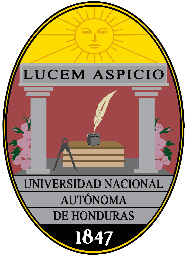
Researcher:** Sheryl Nicole Ruiz
**Public Health Master Student at Lund University | Bachelor of Science in Nursing**
**Cell phone:** (+46) 7######## **Email:** [s#######@student.lu.se](mailto:s)

**Research Study:** Explore the Feasibility of Implementing a Palliative Care Training Program for Non-Health Caregivers in Honduras

**Guide for a Semi-Structured Interview Guide for Palliative Care Experts**

**Experiences:**

1. What challenges have you faced while providing palliative care to patients? Explain
2. How did you overcome these challenges?
3. How would you describe policymakers' and government agencies' support and commitment to palliative care initiatives in Honduras?
4. How would you describe the cultural attitudes toward palliative care in Honduras?
5. Have you been involved in or are you aware of any previous initiatives in Honduras aimed at training non-health caregivers about palliative care? If so, please describe your role or initiative, as well as the outcomes.

PALLIATIVE CARE EXPERTS

**Opinions:**

1. Do you believe that educating non-health caregivers about palliative care is important or useful? Explain.
2. Do you believe that non-health caregivers are best suited to provide palliative care? Explain.
3. What skills or subjects related to palliative care should a non-health caregiver acquire or learn? Explain.
4. How do you think this non-health caregiver training program should be delivered (e.g., manuals, online training, groups in healthcare centers, personalized individual training in healthcare centers, training at home, or others)?
5. What are the challenges you see in developing a home palliative care training program for non-health caregivers in Honduras?
6. Do you believe that a palliative care training program for non-health caregivers is replicable on a national scale?
7. Do you believe that if the family members of patients were given the opportunity to train on this topic, they would accept it? Explain.

**Feelings:**

1. Has your family had any experience with palliative care?
2. If so, did your family member receive palliative care at home from non-healthcare caregivers? Describe your experience**.**

**Knowledge:**

PALLIATIVE CARE EXPERTS

1. What is palliative care?
2. Who should be given palliative care?
3. Who should be in charge of palliative care?

PALLIATIVE CARE EXPERTS

**Research Study:** Explore the Feasibility of Implementing a Palliative Care Training Program for Non-Health Caregivers in Honduras

**Informed Consent for Decision Makers**

You are cordially invited to take part in an interview for this palliative care study. However, before you decide to participate, you should carefully read this form to understand the purpose of the study.

The goal of this study is to gather information on the level of interest and willingness to effectively support and integrate a potential program in Honduras, as well as to identify challenges and opportunities associated with the program's development, through a single interview. The study's findings may be useful for future decision-making in improving palliative care services in Honduras.

Your participation in this research will be entirely voluntary. If you agree to take part, you will sign the informed consent form and be given a copy. The researcher will interview you and record your responses with an audio recorder, which she will later transcribe for the study's analysis in a diary to which only the researcher will have access. The interview may last up to an hour and a half, depending on your comfort level, and will take place in person at a location of your choice. You may take breaks as needed during the interview.

DECISION MAKERS

Certain questions about the subject of study may be personal in nature and cause discomfort. If this occurs, you have the right to withdraw from the research at any time, if you choose, or to refuse to answer questions. Data unrelated to the research questions will not be gathered.

Do you agree to take part in the research?

I have read and understand the information on this form and agree to take part in this research.

**Full Name of Participant:__________________ Date: __________________ (DD/MM/YYYY)
Signature: _________________________________ Fingerprint (if necessary): ________________**


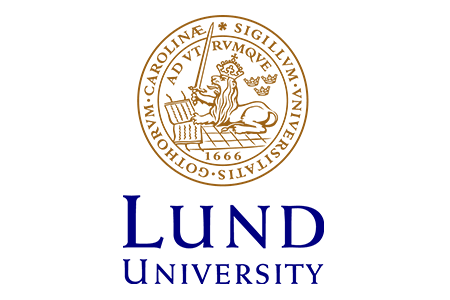
**
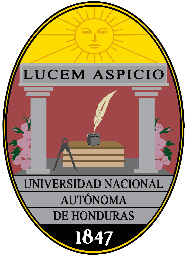
Researcher:** Sheryl Nicole Ruiz
**Public Health Master Student at Lund University | Bachelor of Science in Nursing**
**Cell phone:** (+46) 7######## **Email:** [s#######@student.lu.se](mailto:s)

**Research Study:** Explore the Feasibility of Implementing a Palliative Care Training Program for Non-Health Caregivers in Honduras

**Guide for a Semi-Structured Interview Guide for Decision Makers**

**Experiences:**

1. Have you ever dealt with palliative care issues in your role? If you answered yes, please explain.
2. How would you describe decisionmakers’ support for palliative care initiatives in Honduras?
3. How would you describe the cultural attitudes toward palliative care in Honduras?
4. Have you been involved in or heard of any previous initiatives in Honduras aimed at training non-health caregivers about palliative care? If so, please describe your role or initiative, as well as the outcomes.

DECISION MAKERS

**Opinions:**

1. Do you believe that educating non-health caregivers about palliative care is important or useful? Explain.
2. Do you believe that non-health caregivers are best suited to provide palliative care? Explain.
3. What skills should a non-health caregiver acquire? Explain.
4. How do you think this non-health caregiver training program should be delivered (e.g., manuals, online training, groups in healthcare centers, personalized individual training in healthcare centers, training at home, or others)?
5. What are the challenges you see in developing a home palliative care training program for non-health caregivers in Honduras?
6. Do you believe that a palliative care training program for non-health caregivers is replicable on a national scale?
7. Do you believe that if the family members of patients were given the opportunity to train on this topic, they would accept it? Explain.

**Feelings:**

1. Has your family had any experience with palliative care?
2. If so, did your family member receive palliative care at home from non-healthcare caregivers? Describe your experience.
3. If you had to apply palliative care in the future, would you like to have training in this type of care? Explain
